# Study on the Utilization of Waste Thermoset Glass Fiber-Reinforced Polymer in Normal Strength Concrete and Controlled Low Strength Material

**DOI:** 10.3390/ma16093552

**Published:** 2023-05-05

**Authors:** Yeou-Fong Li, Yi-Wei Hsu, Jin-Yuan Syu, Bian-Yu Chen, Bo Song

**Affiliations:** 1 Department of Civil Engineering, National Taipei University of Technology, Taipei 10608, Taiwan; s103322034@mail1.ncnu.edu.tw (Y.-W.H.);; 2 Department of Civil Engineering, University of Science and Technology Beijing, Beijing 100083, China

**Keywords:** thermoset glass fiber-reinforced polymer, waste, concrete, controlled low strength material

## Abstract

Thermoset glass fiber-reinforced polymers (GFRP) have been widely used in manufacturing and construction for nearly half a century, but the large amount of waste produced by this material is difficult to dispose of. In an effort to address this issue, this research investigates the reuse of thermoset GFRP waste in normal strength concrete (NSC) and controlled low-strength materials (CLSM). The mechanical performance and workability of the resulting concrete were also evaluated. To prepare the concrete specimens, the thermoset GFRP waste was first pulverized into granular pieces, which were then mixed with cement, fly ash, and water to form cylindrical concrete specimens. The results showed that when the proportion of thermoset GFRP waste aggregate in the concrete increased, the compressive strengths of NSC and CLSM would decrease. However, when incorporating 5% GFRP waste into CLSM, the compressive strength was 7% higher than concrete without GFRP. However, the workability of CLSM could be improved to meet engineering standards by adding an appropriate amount of superplasticizer. This finding suggests that the use of various combinations of proportions in the mixture during production could allow for the production of CLSM with different compressive strength needs. In addition, the use of recycled thermoset GFRP waste as a new aggregate replacement for traditional aggregates in CLSM was found to be a more sustainable alternative to the current CLSM combinations used in the market.

## 1. Introduction

Many resources are directly discarded as soon as there is no pressing demand, resulting in the consumption of a large amount of the earth’s resources and energy consumption in manufacturing. This mode of consumption is referred to as the “linear economy,” in which products are manufactured from raw materials and then discarded over time. The improved processing technology has made it possible to mass produce products, and as a result, waste materials or consumables that are discarded during the process or frequently replaced due to obsolescence are generated. The considerable amount of resulting waste could cause permanent damage to the earth. Incinerating or burying these wastes along with general garbage not only affects the environment, but also lowers the quality of life.

The circular economy (CE) is a model of production and consumption that involves remanufacturing, reprocessing, and recycling materials within the production process [1]. This approach to waste management enables the production of goods with minimal waste and helps conserve natural resources. To effectively utilize the limited resources on earth and reduce the impact of waste on the environment, CE, which differs from linear economic consumption behavior, should be emphasized.

In the last few decades, fiber-reinforced polymer (FRP) composites have replaced metallic materials in many areas, such as aerospace engineering, construction engineering, automotive engineering, recreational sports equipment, and wind energy engineering [2,3,4]. The advantages of FRP are its high strength, light weight, acid and alkali resistance, and high weather resistance. The components of FRP composites are fibers and resins. The fibers play a crucial role in determining the physical properties of the composites, and the most commonly used fibers are carbon fibers, glass fibers, and aramid fibers. The resin can be classified as thermoplastic and thermosetting forms. A report conducted by the European Composites Industry Association in 2015 indicated that glass fiber reinforced polymer (GFRP) composites, whose base material is thermosetting resin, constitute a significant portion of the FRP composites industry, compromising more than 95% of all FRP composites [5], and extensive research has been undertaken on the mechanical behaviors of GFRP composite materials with different resin matrices [6,7,8,9,10,11,12].

Despite the benefits FRP composite provides, its end-of-life management poses many negative consequences. When fed into the incinerator for combustion, waste thermosetting composites, unlike thermoplastic composites, which are re-moldable and recyclable, might release a large amount of toxic gas while burning and pose a threat to the environment and human life, and the incineration ash might also pollute the soil. When buried in landfills for disposal, the waste not only affects the operational function of the soil and the living environment of the nearby residents but also causes harm to the land’s resources. As a result, many countries have enforced regulations prohibiting the disposal of thermosetting FRP waste in such a manner.

A major source of waste FRP composite is from decommissioned wind turbine blades, of which between 80% and 90% of the blade mass is composite material [13], and the dominant composite material in the blades is GFRP. The proliferation of wind turbines in recent years has been driven by the growing demand for renewable energy sources. As demand for wind energy increases, the size of wind turbines and the quantity of materials needed for the blades also grow [14]. Given the average service life of wind turbine blades, which ranges from 20 to 25 years [14,15], it was estimated that by 2050, the total amount of waste wind turbine blades on the globe will reach 43 million tons [16].

Currently, as mentioned before, several options exist for managing GFRP waste: disposal in a landfill, incineration, or recycling [17]. However, there are limitations and drawbacks to these methods. Despite being a non-biodegradable material, it was observed that the vast majority of GFRP waste in the United States was disposed of in landfills [18]. There has been a continuing increase in environmental awareness globally, leading to efforts by various countries to decrease the amount of waste being sent to landfills. To support these efforts, laws have been implemented to protect land usage. For example, in June 2005, the German government implemented a landfill disposal ban on GFRP [15]. Unlike landfill disposal, incineration is a practice that reduces the volume of wind turbine blades, and the combustion of epoxy resin could contribute to energy recovery. However, this is not a feasible option for managing GFRP waste, as GFRP is incombustible. Additionally, the emissions produced during the combustion of epoxy resin may include harmful byproducts due to the presence of inorganic resin [19,20,21].

Recycling GFRP waste involves recovering, with the aim of repurposing, fibers and resin from waste materials through mechanical, thermal, and chemical processes [22]. Mechanical recycling involves shredding GFRP waste materials into powders and smaller coarse particles. It is a more environmentally friendly and relatively simpler method compared with the abovementioned recycling processes. In addition, mechanical recycling requires the least recycling energy consumption at 0.27–3.03 MJ/kg [23]. However, this method primarily focuses on the production of powders, which can be used as energy sources [24]. The repurposing of the fiber materials in this method is difficult, as crushing GFRP materials would lead to significant degradation of the fibers’ mechanical properties [25].

Chemical recycling of GFRP composite materials involves the use of a solvent to dissolve the polymer matrix and convert the polymer into monomer while the fibers stay intact [26,27] and retain their original mechanical properties, and the recovered monomer can be used to produce new polymers. Chemical recycling seems to support a circular economy that allows for the circulation of the recycled material [28]. However, chemical recycling usually requires higher temperatures and high pressure, resulting in the highest recycling energy consumption at 63–91 MJ/kg among all three recycling methods [23]; moreover, the solvents used are usually toxic.

Thermal recycling utilizes heat energy at an operating temperature of 450 °C to 700 °C to separate the fiber and the resin from the composite material. Between combustion, fluidized bed, and pyrolysis, pyrolysis thermal recycling is the most commonly used in commercial applications [24], and, unlike chemical recycling, thermal recycling has the advantage of not utilizing chemical agents and not producing harmful chemicals. However, the retrieved fibers do not retain their original mechanical strength, usually decreasing by 50% to 64% [28]. The mechanical properties of composites fabricated with glass fiber recovered from pyrolysis have been found to be significantly degraded in comparison to those fabricated with original fibers [9,29]. The various methods of managing GFRP waste mentioned above each have their own advantages and drawbacks. These drawbacks may include the degradation of fiber mechanical properties, a long degrading time, high energy consumption, and the production of toxic byproducts.

To achieve sustainable green development, utilizing waste as aggregate in cement-based materials for civil engineering construction is one of the possible ways to solve the global waste generation problem and conserve natural resources. Research exploring such waste utilization in cement, including the use of waste glass powder, bottom ash from burning plants, and fibers, remains a popular topic in this field [30,31,32,33,34,35]. The waste thermoset GFRP composite was used as aggregate and filler replacement for concrete, mortar, and bitumen-reinforced concrete, and their mechanical performance, durability performance, and underlying mechanisms were discussed [36,37,38,39,40,41]. Controlled low-strength material (CLSM) is a highly flowable cementitious material with self-consolidating properties. It can be used for structural fills, road pavement foundations, and conduit fills. Moreover, it can be applied in small areas where compaction equipment cannot be accessed. Because of the small disparity between its strength and the soil compaction strength, the area can be easily excavated during reconstruction. The feasibility of CLSM production using ponded ash, fly ash, circulating fluidized bed combustion ash, and excavated soil wastes as full replacements for Portland cement and alkaline activation in CLSM was evaluated [42,43].

The basic properties and the content of the waste materials, as well as the amount of water in the mix design, play a dominant role in determining the plastic properties of CLSM. The plastic properties of CLSM, such as flowability, bleeding, segregation, and hardening time, are found to be inter-related. With proper design of the waste materials, it is possible to produce CLSM with acceptable plastic properties [44,45]. The recycled product from liquid crystal display (LCD) glass waste was used to replace sand with different weight ratios in the production of CLSM, and the resulting compressive strength, supersonic strength, electrical resistivity, and permeability ratio were tested. The results showed that adding the proper amount of LCD glass waste into CLSM would meet the corresponding engineering requirements [46]. The feasibility and applicability of incorporating solid wastes from paper mills into CLSM were also studied. Paper sludge was treated as a fibrous admixture, fly ash was used as a substitute for cement, and bottom ash was added by partially replacing the fine aggregate. The results showed that solid wastes/byproducts from paper mills could be effectively used in the production of CLSM mixtures with desired performances [47]. Using stainless steel-reducing slag as the cement substitute in the production of soil-based CLSM is also a solution to reduce solid wastes/byproducts. The test results showed that increasing the stainless steel-reducing slag substitution level would improve workability, extend the setting time, decrease pulse velocity, and reduce the compressive strength of CLSM gradually [48]. The sodium hydroxide (NaOH) solution was used to activate fly ash and ground granulated blast-furnace slag with the addition of different ratios of wastewater treatment sludge to produce the alkali-activated CLSM [49].

To reduce the negative impacts and costs of GFRP production, countries worldwide are developing recycling and repurposing technologies. This supports the transition to a more sustainable GFRP manufacturing industry and a circular economy. While previous studies have investigated the use of crushed GFRP waste as a replacement for fine aggregate in concrete and examined the production of CLSM with ponded ash, fly ash, circulating fluidized bed combustion ash, and excavated soil waste as a full replacement of fine aggregate, the possibility of incorporating crushed GFRP waste into CLSM while meeting engineering standards requires further exploration. This study aims to investigate the feasibility of integrating waste thermosetting GFRP composite crushed materials (referred to hereafter as “GFRP waste”) into CLSM. By exploring this approach, we hope to identify potential opportunities to further reduce the environmental impact of GFRP production while still meeting engineering standards for CLSM.

## 2. Materials

### 2.1. GFRP Waste

**Figure 1:**
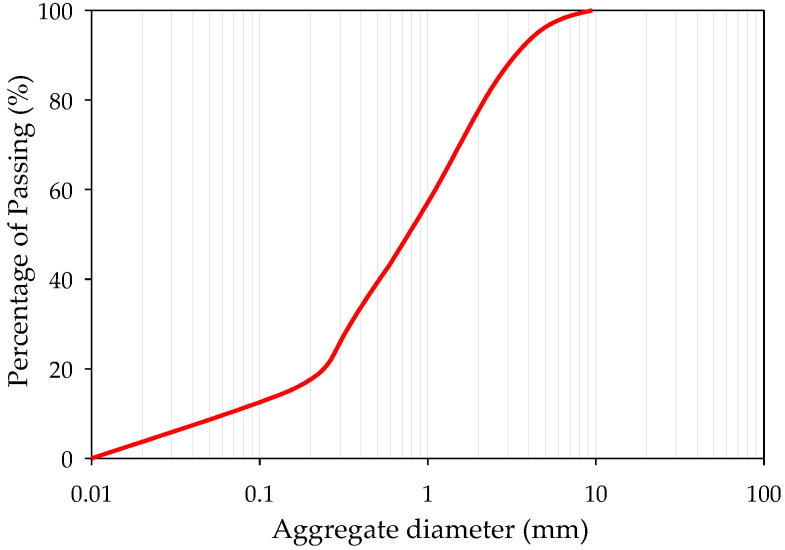
Sieve analysis of GFRP waste.

**Figure 2:**
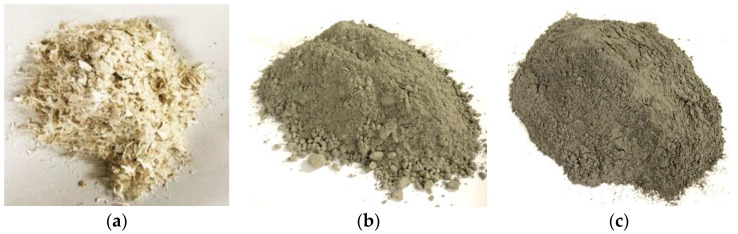
CLSM materials: (**a**) crushed thermoset GFRP waste; (**b**) type I Portland cement; (**c**) fly ash.

The GFRP waste was obtained from the offcut of the sheet molding compounds (SMC) process provided by Nan-Ya Plastics Corporation, New Taipei City, Taiwan. The composition of the GFRP waste is shown in Table 1. Parts of the sand used in concrete were substituted by the crushed GFRP waste in this study. The fineness modulus (F.M.) of the GFRP waste was 2.76, as calculated and presented in Table 2. The sieve analysis of the GFRP waste is shown in Figure 1, and the GFRP waste is shown in Figure 2a.

### 2.2. Cement

In this study, type I Portland cement was used to produce normal strength concrete and CLSM, as shown in Figure 2b.

### 2.3. Fly Ash

In this study, the fly ash was provided by Advanced-Tek Systems Co., Ltd. (Taipei, Taiwan), as shown in Figure 2c; the composition of fly ash is shown in Table 3.

### 2.4. Aggregates

**Figure 3:**
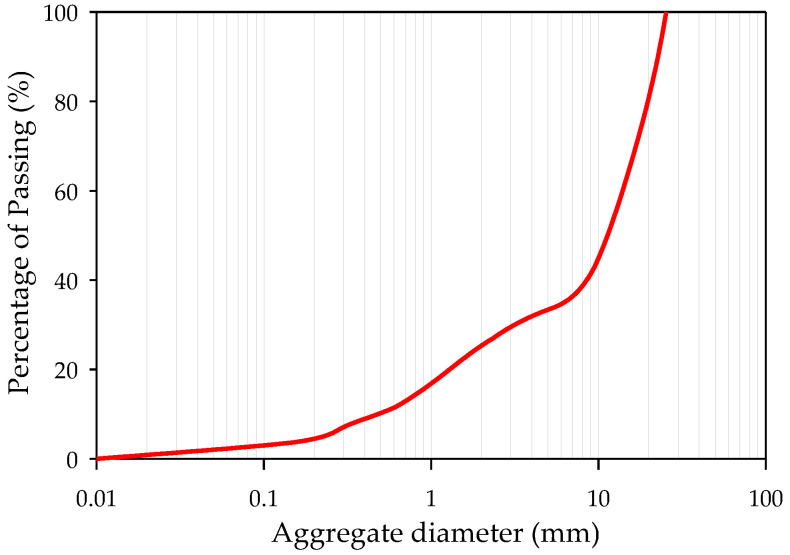
Sieve analysis of aggregates.

In this study, the aggregates were provided by Goldsun Co., Ltd. (Taipei, Taiwan). The fineness modulus of aggregates was calculated to be 4.99, as shown in Table 4. Figure 3 shows the sieve analysis of aggregates.

### 2.5. Superplasticizer

The polycarboxylic-ether-based superplasticizer Glenium 51 (Yo-Rich Co., Ltd., Taoyuan, Taiwan) was used in this study to reduce the mixing water content, prevent early flocculation or stiffening, and increase the workability of the CLSM mix. The addition of superplasticizer was below 0.01% (*w*/*w*).

## 3. Test Methods

In this study, two types of cylindrical concrete specimens with a diameter of 10 cm and a height of 20 cm were used: normal strength concrete (compressive strength 20~40 MPa) and CLSM (compressive strength < 8.4 MPa). The two concrete mixes were prepared with varying proportions of crushed GFRP waste materials. The GFRP waste materials were incorporated for two different purposes. The first was to replace a proportion of the fine aggregates in the cement mix, and the second was to function as additional binding agents in the cement mix. After finding the ideal range of CLSM proportions, a superplasticizer and additional water were added and then mixed with water to enhance the workability and early strength.

### 3.1. Preparation of Normal Strength Concrete Specimens

A twin shaft paddle mixer was used to make concrete specimens, which were manufactured according to ASTM C192 [50]. In the production of normal strength concrete, after the cement, the fine aggregate (sand), and the GFRP waste materials were weighed, the mixture was put into the agitator kettle and mixed evenly. Then, water was added to enable the stirring of the agglomerates at the bottom or edge of the kettle. Finally, the coarse aggregates were added and mixed evenly. The evenly mixed concrete was then divided into three layers and rammed into a paper mold with a diameter of 10 cm and a height of 20 cm.

Table 5 shows the design proportion of the normal strength concrete. The GFRP waste materials were used as the filler and were added from 0% to 20% with an equal difference of 5%. Similarly, the GFRP waste materials were used as a substitute for fine aggregates (sand) and were added from 0% to 20% with an equal difference of 5%. The density of the crushed GFRP waste materials was approximately 1.8 g/cm^3^ and that of the fine aggregate was approximately 2.7 g/cm^3^. To maintain the same volume of the fine aggregates, the weight of sand was replaced by 1.5 times the weight of GFRP waste materials. The specific weight of the normal strength concrete was approximately 2200 kg/m^3^. In this subsection, specimen B serves as the benchmark for normal strength concrete. Specimens labeled “G” are samples with a certain percentage of GFRP waste added as additional material, while specimens labeled “RG” have the fine aggregates in the mix replaced by a certain percentage of GFRP waste. The numbers following the letters “G” and “RG” indicate the percentage of GFRP waste added or used as a replacement for fine aggregates, respectively. The strength of the two groups, specimens labeled “G” and “RG”, is then compared to that of the benchmark specimen, specimen B.

### 3.2. Preparation of CLSM Specimens

The CLSM resembles that of normal strength concrete but has higher workability caused by additional fly ash and uses a smaller amount of cement. In terms of the coarse aggregates, only 3/8″ gravels were added to the CLSM. The following study illustrated the procedure for adding the CLSM superplasticizer, which was mixed with water before being added to the mixed granular materials.

Table 6 shows the design proportion of the CLSM. GFRP waste with an additional weight of 0 g, 100 g, and 200 g, was used to replace part of the sand in this part of the test. The sum of the weights of cement and fly ash was 200 g. A total weight of 200 g of cement decreased progressively in intervals of 20 g until it reached 100 g, while a total weight of 0 g of fly ash increased progressively by 20 g until it reached 100 g. The specific weight of the CLSM was approximately 1900 kg/m^3^. The naming convention is as follows: specimen C100G100 referred to the specimen with a cement to CLSM ratio of 100 and a proportion of GFRP waste to CLSM of 100. Additionally, specimen C100BG0 referred to the benchmark specimen whose cement to CLSM proportion was 100 and without GFRP waste. In this section, the amount of additional water was determined based on the workability of the CLSM slurry because of the influence of glass fiber in GFRP waste.

### 3.3. Compressive Strength Test

**Figure 4:**
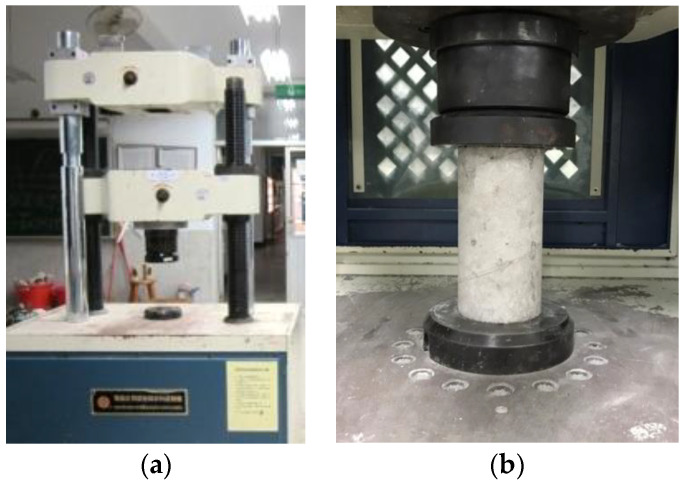
Compressive test setup: (**a**) universal testing machine HT-9501; (**b**) cylindrical concrete specimen.

The compressive test was conducted using a universal testing machine (HT-9501 Series. Hong-Ta, Taiwan) with a load cell (WF 17120, Wykeham Farrance, Milan, Italy), as shown in Figure 4a. The compression test of the normal concrete was conducted as per CNS 1232 [51] (similar to ASTM C39/39M-04a [52]) with 100 kN/min as the rate of compression. The compression test of the CLSM was performed per CNS 15865 [53] (similar to ASTM D4832-16e1 [54]), with the rate of compression being 5 kN/min. The cylindrical concrete specimen for the compressive test is shown in Figure 4b.

## 4. Results and Discussion

This subsection discusses the results from the compressive strength test conducted on normal strength concrete and CLSM with varying amounts of GFRP waste incorporated.

### 4.1. Compressive Test Results of the Normal Strength Concrete

The purpose of this subsection is to investigate the effect of adding GFRP waste material to normal strength concrete on its compressive strength, and examine the impact of substituting a portion of fine aggregates with GFRP waste materials.

**Figure 5:**
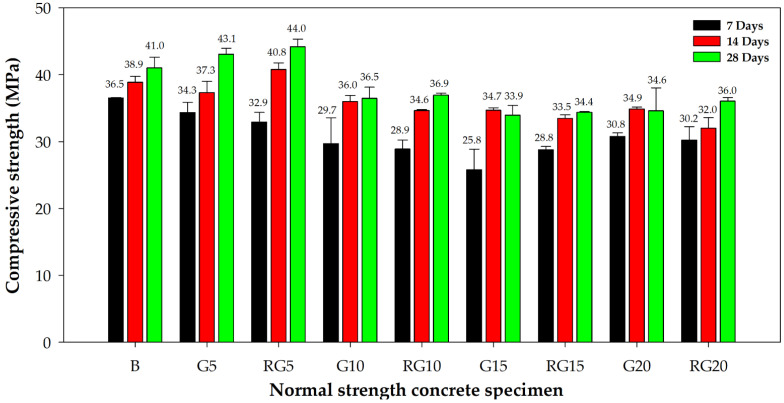
Average compressive strength of the normal strength concrete at 7, 14, and 28 days.

Figure 5 shows the average compressive strength of the specimens at the ages of 7, 14, and 28 days. The result illustrates the trend that increasing the amount of waste thermosetting glass fibers would decrease the compressive strength of normal strength concrete by 10–25%. However, specimens G5 and RG5 attained higher compressive strengths than the benchmark at the age of 28 days, indicating that incorporating the optimal amount of fiber could in fact increase the performance of the material.

It could be observed in Figure 5 that the strength differences between specimens with additional GFRP waste, labeled ‘R’, and specimens with GFRP waste replacing part of the fine aggregate, labeled ‘GR’, were within the experimental error. This was an indication that replacing part of the natural granular materials with GFRP waste was feasible, as such a substitution had minimal effect on the compressive strength.

### 4.2. Compression Test Results of the CLSM

This subsection aims to explore the feasibility of producing CLSM in different proportions that meet different design strengths. Table 7 shows the average compressive strength of the specimens at 7, 14, and 28 days, respectively.

**Figure 6:**
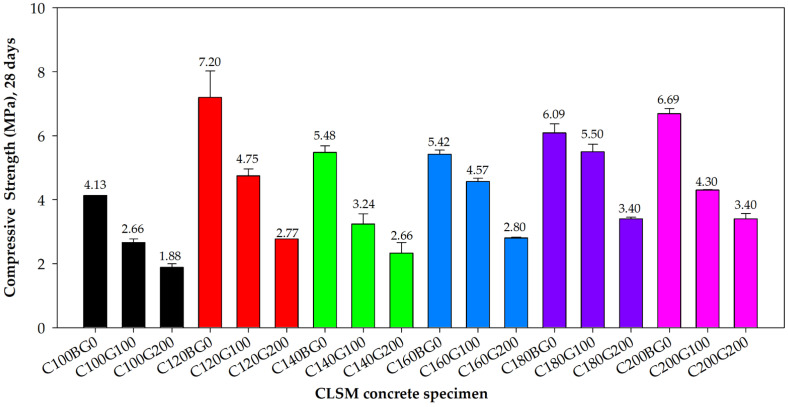
Average compressive strength of the CLSM at 28 days.

**Figure 7:**
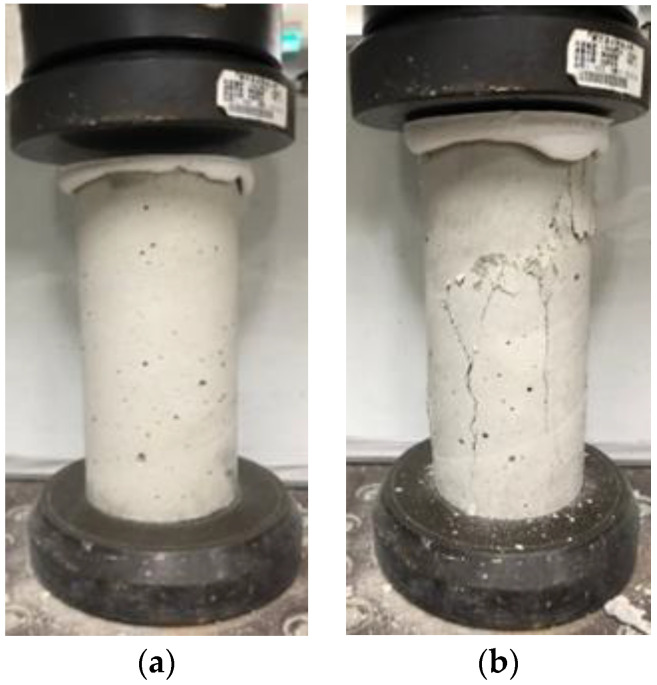
Photos of CLSM specimens under compressive tests: (**a**) before failure; (**b**) after failure.

A series of compressive strength intervals were obtained from the above test results on the CLSM compressive strength with different proportions. As per ASTM D4832-16e1 [54], the mix design is typically based on 28-day strengths; thus, the average compressive strength of different specimens at 28 days is shown in Figure 6. Figure 7a,b shows the photos of CLSM specimens under compressive test before and after failure, respectively.

As seen from the above results, the compressive strengths of all the designs proportional to the age of 28 days all meet the ASTM D4832-16e1 standard, which does not exceed 8.4 MPa [54]. Therefore, by adjusting the cement to CLSM and the GFRP to CLSM ratios, different design strengths could be achieved.

## 5. On-Site Application of CLSM

The purpose of this subsection is to investigate the on-site applicability of the CLSM incorporating GFRP waste. As discovered in the previous subsection, all specimens met the ASTM D4832-16e1 standard. Therefore, in this subsection, two of the acceptable specimens, C140G100 and C140BG0, were selected for slump flow and Kelly Ball tests. To improve the workability, proportional superplasticizer and extra mixed water were added. Table 8 lists the benchmark and one of the design proportions, namely C140G100, and the letter “S” in front of C140BG0 and C140G100 denotes the specimens with superplasticizer and additional water added.

### 5.1. CLSM Slump Flow Test for On-Site Practice

**Figure 8:**
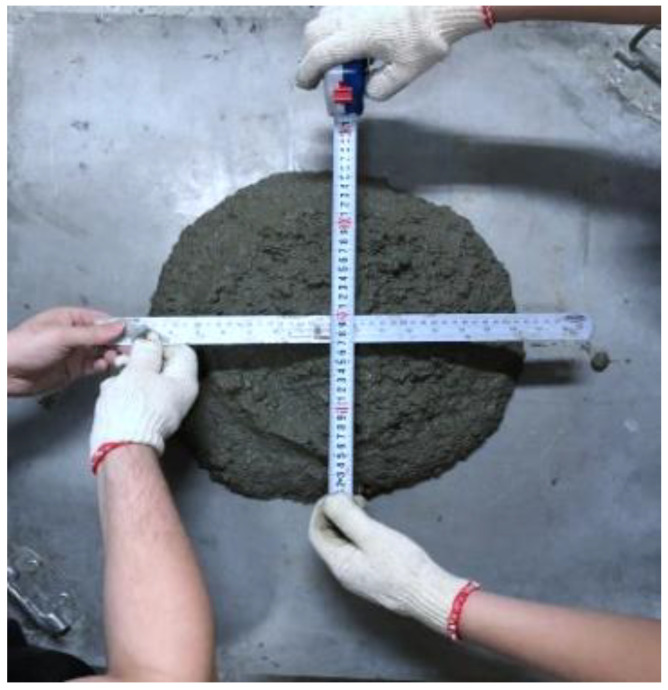
Slump flow test of the CLSM.

According to ASTM D6103/ D6103M-17 [55], an open-ended cylindrical mold of concrete mix was placed on a flat, level surface and filled with fresh CLSM mix. The cylindrical mold with a height of 30 cm was raised quickly so the CLSM would flow into a patty. Then, the longest diameter of the patty as well as the diameter in a direction orthogonal to the longest diameter were measured with a precision of 1 mm. The average diameter of the patty was determined, and the measuring photo is shown in Figure 8.

### 5.2. Method of Drop Ball Testing for the Timing for Adding Weight to CLSM for On-Site Practice

**Figure 9:**
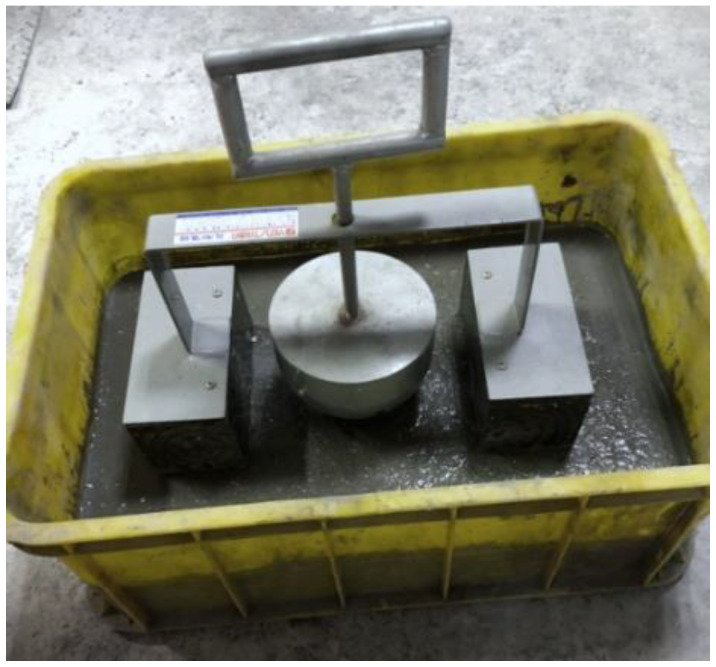
The Kelly ball apparatus for measuring the diameter of indentation of the CLSM.

According to ASTM D6024/D6024M-16 [56], the test method was designed to evaluate the ability of CLSM to load through repeated dropping of a weight onto the material in place. This ability is quantified by the diameter of the indentation. As per ASTM D6024/D6024M-16, the subcommittee does not have knowledge of any published data or studies that establish specific acceptance criteria for this diameter. However, subcommittee members who have experience applying the method have reported that a common criterion in utility applications, such as trench backfill, is 75 mm [56]. A half-spherical metal object fell from a fixed distance onto the surface of the in-place CLSM specimen five times, as shown in Figure 9. The indentation is inspected for any free water brought to the surface from the impact. The purpose of the test was to ensure the load-bearing capacity of the CLSM when heavy objects are temporarily added on top.

To test the CLSM workability, the Kelly ball was used to examine the drop strength within a day and the compressive strength at the age of 28 days, the results of which are shown in Table 9, Table 10 and Table 11. The diameter of the indentation was about 70 mm, and there was no obvious presence of surface water within the dent, which suggested that using GFRP waste in producing CLSM was feasible for producing on-site CLSM that conforms to ASTM standards.

Specimen SC140G100 represented one of the ideal proportions in this study, as it met the abovementioned ASTM standards. Adding proportional superplasticizer and mixed water improved the fluidity and compressive strength of the CLSM at 28 days of age. The drop strength within one day also met the ASTM specification, which suggested feasibility for on-site practice. Test results show that GRPF waste is feasible to incorporate into CLSM for engineering applications.

## 6. Conclusions

The objective of this research was to identify and propose an environmentally sustainable and innovative solution for repurposing the substantial upcoming GFRP waste. This study examined the re-utilization of thermoset GFRP waste in normal strength concrete and CLSM. Based on the findings, the following conclusions can be inferred:

1. Inclusion of more than 5% GFRP waste into normal strength concrete, either incorporated as additional fine aggregate or as a replacement of fine aggregate, resulted in a decrease in the compressive strength of the concrete by a range of 10% to 25%.
2. In normal strength concrete, partially replacing natural granular materials with GFRP waste and incorporating GFRP waste as additional aggregate do not show significant differences in their compressive strengths. Additionally, the study found that replacing fine aggregate with 5% GFRP waste led to concrete with a final compressive strength that was 7% higher than concrete without GFRP.
3. When the proportion of GFRP waste is increased in CLSM, there is a corresponding decrease in the compressive strength and workability of the concrete. As GFRP waste is composed of recycled sheet materials, it tends to hinder the concrete’s workability when mixed. To improve fluidity and reduce the amount of cement needed, the addition of fly ash may be beneficial.
4. By adjusting the ratios of cement-to-CLSM and GFRP waste-to-CLSM, CLSM can be produced to meet various design strength requirements.
5. GFRP waste-incorporated CLSM, with the standardized addition of proportional superplasticizer and additional mixed water, is feasible for onsite applications as it meets the related ASTM standards.

## Figures and Tables

**Table 1:** The composition of the GFRP waste.

Component	Weight Percentage (%)
CaCO_3_	45~55
Glass Fiber	20~23
Resin	26~32
Other Additives	≤6

**Table 2:** The fineness modulus of the GFRP waste.

Sieve No.	Sieve Size (mm)	Weight (g)	Percentage of Individual Fraction Retained by Weight	Cumulative Percentage Retained by Weight
4	4.75	80.5	4.32	4.32
8	2.36	250.2	13.44	17.76
16	1.18	380.0	20.41	38.17
30	0.60	340.5	18.29	56.46
50	0.30	330.0	17.72	74.18
100	0.15	200.2	10.75	84.93
Pan	<0.15	280.5	15.07	(100)
Total	-	1861.9	100.00	276
				F.M. = 2.76

**Table 3:** The composition of fly ash.

Component	Weight Percentage (%)
SiO_2_	62.9
Al_2_O_3_	24.0
Fe_2_O_3_	3.92
SO_3_	0.24
Water	0.06
Others	6.56
Loss on ignition	2.32

**Table 4:** Fineness modulus of aggregates.

Sieve No.	Sieve Size (mm)	Weight Retained (g)	Percent Retained (%)	Cumulative Percent Retained (%)
4	4.75	1983	66.93	66.93
8	2.36	179	6.04	72.97
16	1.18	242	8.17	81.13
30	0.60	220	7.42	88.56
50	0.30	128	4.32	92.88
100	0.15	102	3.44	96.32
Pan		109	3.68	(100.00)
Total		2963	100	498.79
				F.M. = 4.99

**Table 5:** Design proportion of the normal strength concrete (unit: g).

Specimen	Cement	GFRP Waste	Sand	3/8″ Gravel	3/4″ Gravel	Water
B	100	0	100	133	67	45
G5		5	100			
RG5		5	92.5			
G10		10	100			
RG10		10	85			
G15		15	100			
RG15		15	77.5			
G20		20	100			
RG20		20	70			

**Table 6:** Design proportion of the CLSM (unit: g).

Specimen	Cement	Fly Ash	GFRP Waste	Sand	3/8″ Gravel	Water
C100BG0	100	100	0	1343	400	200
C100G100			100	1193		240
C100G200			200	1043		300
C120BG0	120	80	0	1343		200
C120G100			100	1193		240
C120G200			200	1043		300
C140BG0	140	60	0	1343		240
C140G100			100	1193		280
C140G200			200	1043		340
C160BG0	160	40	0	1343		240
C160G100			100	1193		280
C160G200			200	1043		340
C180BG0	180	20	0	1343		240
C180G100			100	1193		280
C180G200			200	1043		340
C200BG0	200	0	0	1343		240
C200G100			100	1193		280
C200G200			200	1043		340

**Table 7:** Average compressive strength of the CLSM after different curing days.

Specimens	Average Strength (MPa) at 7 Days	Average Strength (MPa) at 14 Days	Average Strength (MPa) at 28 Days
C100BG0	1.46	1.67	4.13
C100G100	1.02	1.89	2.66
C100G200	0.95	1.21	1.88
C120BG0	3.54	5.22	7.20
C120G100	2.20	3.17	4.75
C120G200	1.20	1.52	2.77
C140BG0	3.44	3.67	5.48
C140G100	1.98	2.49	3.24
C140G200	1.11	1.60	2.33
C160BG0	3.47	4.25	5.42
C160G100	2.70	4.00	4.57
C160G200	1.57	2.24	2.80
C180BG0	4.25	5.29	6.09
C180G100	3.54	4.80	5.50
C180G200	1.90	2.67	3.40
C200BG0	4.84	6.50	6.69
C200G100	3.07	4.15	4.30
C200G200	2.32	3.21	3.40

**Table 8:** Formula design of the on-site CLSM (unit: g).

Specimen	Cement	GFRP Waste	Fly Ash	3/8″ Gravel	Sand	Water	Superplasticizer
SC140BG0	140	-	60	400	1343	280	2
SC140G100		100			1193	310	

**Table 9:** Flow consistency of the on-site CLSM.

Specimen	Slump (mm)	Flow (mm) *
SC140BG0	230	415
SC140G100	218	405

* ASTM D6103 standard is ≥200 mm.

**Table 10:** Drop strength of the on-site CLSM (1 day).

Specimen	Number of Drop	Diameter of Drop (mm)
SC140BG0	5	52
SC140G100	5	70

**Table 11:** Compressive strength of the on-site CLSM.

Specimen	Average Strength (MPa) on the Age of 7 Days	Average Strength (MPa) on the Age of 14 Days	Average Strength (MPa) on the Age of 28 Days *
SC140BG0	2.88	3.94	5.12
SC140G100	1.91	2.48	3.6

* ASTM D4832 standard is ≤8.4 MPa (28 days).

## Data Availability

Not applicable.
